# Comparative analysis of the push-out bond strength of fiber posts: Immediate vs. delayed post-space preparation with two obturation techniques

**DOI:** 10.1371/journal.pone.0333880

**Published:** 2025-10-16

**Authors:** Weilin Long, Xiongjun Xu, Li Tang, Hongwei Jiang, Yihua Huang

**Affiliations:** 1 Department of Stomatology, The Third Affiliated Hospital of Sun Yat-sen University, Guangzhou, Guangdong, P.R. China; 2 Hospital of Stomatology, Guanghua School of Stomatology, Sun Yat-sen University, Guangzhou, Guangdong, P.R. China; 3 Guangdong Provincial Key Laboratory of Stomatology, Guangzhou, Guangdong, P.R. China; Universidade Federal Fluminense, BRAZIL

## Abstract

**Objectives:**

The objective of this study was to assess the impact of immediate and delayed post- space preparation on the push-out bond strength (PBS) of fiber posts by employing two root canal obturation techniques: continuous wave of condensation (CWC) and single-cone (SC) obturation.

**Materials and methods:**

Forty-eight human maxillary premolar teeth were instrumented, and the samples were divided into four groups according to the obturation technique and the time of post-space preparation. SC and CWC underwent immediate post-space preparation; and CWC and SC underwent delayed post-space preparation. The smear layer and dentine tubules from the apical, middle, and cervical regions of the samples were observed via scanning electron microscopy (SEM) and the %Voids_Vol_ of the medium 4-mm fiber posts of each group was calculated via micro-computed tomography (CT). Each post space of the root was subsequently cut into slices, resulting in three 1-mm slices at 3 different depths (apical to the cervical region) and subjected to a push-out test. The failure mode was assessed. The data were analysed via the Shapiro-Wilk test, one-way analysis of variance and Bonferroni correction tests.

**Results:**

In terms of depth from the apical to the cervical region, SC following immediate post-space preparation exhibited greater bond strength than did CWC following delayed post-space preparation. SEM images revealed that the smear layer was completely visible. In the SC with immediate post-space preparation group, the smear layer could be partially removed from the apical, middle and cervical regions of the samples and the outlines of the dentine tubules were visible. The percentage volume of the voids of the medium 4-mm fiber posts of the four groups and the samples in the three directions were not significantly different. No significant differences were observed in the CWC or SC obturation technique regardless of the time of post-space preparation or in immediate or delayed post-space preparation with different obturation techniques.

**Conclusions:**

SC followed by immediate post-space preparation provided better bond strength of fiber posts to intraradicular dentine than did CWC followed by delayed post-space preparation.

## Introduction

The criteria for endodontic success emphasize the importance of proper cleaning, shaping, and obturation of the root canals. Adequate obturation aims to completely fill and seal the entire root canal system, including its ramifications. Achieving high-quality obturation requires the selection of appropriate techniques. Previous research studies have shown that the continuous wave of condensation (CWC) technique is commonly chosen because of its ability to better fill canal irregularities [1]. The advent of Ni-Ti matched-tapered gutta-percha cones, coupled with enhancements in root canal sealer properties, has contributed to the growing adoption of the single-cone (SC) obturation technique in clinical practice. It is considered to have lower technical sensitivity and gentler on the dentin wall [2–4].

Adhesively cemented intraradicular posts become essential in endodontically treated teeth with insufficient coronal structure to retain the restoration. Their function is to distribute occlusal forces evenly along the root canal, thereby safeguarding against tooth fracture [5,6]. Over the past two decades, various techniques and materials have been introduced for restoring endodontically treated teeth [7]. Among these, fiber posts have become increasingly favored over traditional prefabricated or cast metal posts due to their superior physical properties in post-crown restorations. Nevertheless, the primary failure mode observed in fiber posts is debonding of the post, with the bond strength being clinically significant [8–11]. In dental literature, the push-out test has been widely employed because of its precision, reliability, and appropriateness as the most accurate method for measuring post retention [12,13]. Since the SC technique is relatively new, information regarding its potential interference with fiber posts cemented with resin-based cements is still limited.

Beyond the obturation technique, the timing of post space preparation also affect the bond strength of cemented fiber posts. To date, the literature lacks consensus on whether posts should be placed immediately after root canal treatment or delayed until a later stage following obturation. To simplify the procedure and shorten the treatment duration, immediate post-space preparation has gradually gained clinical attention. However, some scholars suggest delaying post space preparation to observe the root canal filling treatment and waiting for the filling material to completely solidify [14]. Conversely, a study indicated that delayed cementation of fiber posts increases their retentive strength [1]. Currently, there is limited evidence to guide clinicians regarding the optimal timing for cementing a fiber post.

Given the clinical importance of preventing fiber posts displacement caused by reduced bond strength to dentin, this study aimed to evaluate the effects of different obturation techniques (CWC and SC) and the timing of post space preparation (immediate vs. delayed) on fiber posts bond strength. The null hypothesis posited that neither the obturation technique nor the post-space preparation timing would negatively affect the bond strength between the fiber post-adhesive cement interface and root dentin.

## Materials and methods

### Specimen preparation

Ethical approval for this study was granted by the Ethics Committee of the Hospital of Stomatology and Guanghua School of Stomatology, Sun Yat-sen University (Approval No. ERC – [2017] −16; Guangzhou, China). Written informed consent was obtained from all participants and/or their legal guardians (for minors under 18 years) who voluntarily donated their extracted teeth for research purposes between June 1, 2018 and February 3, 2022. After obtaining informed consent from all participants, forty-eight permanent human maxillary premolar teeth were collected. These teeth had been extracted for orthodontic treatment at Guanghua School of Stomatology, Affiliated Stomatological Hospital, Sun Yat-sen University. The extracted teeth were stored in saline solution at room temperature to preserve tissue hydration and maintain surface moisture. Teeth with double root canals free of caries, fractures, resorption, or defects were selected. The teeth were decoronated at the cementoenamel junction. The length of the root and the bucco-lingual diameter and proximal‒distal diameter of the cervical region was measured. To standardize techniques and minimize variability, one experienced clinician performed all clinical procedures: root canal preparation, obturation, and fiber posts cementation.

Using M3 rotary instruments (30.04) (M3 Rotary System, United Dental, China), canals were prepared to 0.5 mm short of apex with alternating NaOCl (1%) and saline irrigation (5 mL each). Canals were treated with 17% EDTA (5 mL, 1 min) for smear layer removal, then rinsed with saline (5 mL). Root canals were dried with sterile absorbent paper points and filled with calcium hydroxide paste (Ivoclar Vivadent, Schaan, Liechtenstein) for a two-week intracanal sterilization period. The teeth were subsequently stored at 37°C in 100% humidity. Following sterilization, apical patency was verified using a No. 10 K-file (Dentsply Maillefer, Ballaigues, Switzerland). The calcium hydroxide was then rinsed out with saline solution, and canals were re-dried with paper points.

The root canals were obturated using a size 30.04 taper gutta-percha cone (3M ESPE, Seefeld, Germany) in conjunction with iRoot SP bioceramic sealer (Innovative BioCeramix Inc, Vancouver, BC, Canada). The master gutta-percha cone was first fitted to the prepared canal to verify proper adaptation and tug-back resistance. The obturation protocol involved injecting iRoot SP sealer directly into the canal space followed by coating the master cone with a thin layer of sealer. For the CWC group, continuous wave compaction was performed using a preheated plugger that was gradually advanced through the gutta-percha cone, maintaining a 4-mm apical seal. The coronal portion was subsequently obturated with thermoplasticized gutta-percha using a backfilling technique. In contrast, the SC group underwent single-cone obturation where the master gutta-percha cone was sectioned at the canal orifice level

On the basis of the obturation technique and the timing of post-space preparation, the teeth were randomly allocated into four experimental groups (n = 12): SC and CWC underwent immediate post-space preparation (SC-I and CWC-I), whereas CWC and SC underwent delayed post-space preparation (SC-D and CWC-D).

### SEM imaging

The coronal gutta-percha in the palatal canal was removed using the heating element of the obturation system (Elements Obturation Unit, SybronEndo, Kerr Endodontics, Orange, CA), while maintaining a standardized 4-mm apical seal. Post spaces were then prepared using the corresponding drill from the post-space preparation kit (3M ESPE, Seefeld, Germany) to match the dimensions of the prefabricated fiber posts (color-coded red; 3M ESPE, Seefeld, Germany). Post spaces were washed with 1% NaClO and normal saline between instrumentation, irrigated with 5 mL of saline solution and dried with absorbent paper points according to the manufacturer’s instructions.. Two random samples were selected from each experimental group to assess the dentin tubule openings in different regions of the root surface. Surface topographic changes were analyzed using scanning electron microscopy (SEM) at ×2,000 magnification, with both the operators and analysts blinded to the group assignments.

### Fiber posts cementation

Dentin adhesion was achieved via Scotchbond^TM^ Universal Adhesive (3M ESPE, Seefeld, Germany). Following the manufacturer’s instructions, the post was cleaned with alcohol, and Scotchbond^TM^ Universal Adhesive was applied to both the post space wall and the surface of the post. Paper points were employed to dry the post space, and gas was used to ensure even drying of the adhesive. RelyX^TM^ Ultimate Adhesive Resin Cement (3M ESPE, Seefeld, Germany) was then injected into the post space, and the post (coloured red) was inserted for 6 min for polymerization. Specimens from the delayed post-space preparation subgroups (CWC-D and SC-D) were conditioned in a controlled environment (37°C, 100% relative humidity) for 1 week.

### Micro-CT analysis

Following specimen preparation, high-resolution micro-computed tomography (μCT) was performed to quantitatively assess the volumetric distribution of filling materials and void fractions within the 4-mm sections of the medium-sized fiber posts. Micro-CT scans were acquired via a micro-CT scanner (micro-CT uCT50, Scanco Medical AG, Basserdorf, Switzerland) with scanning parameters of 8 W, 70 kV, 114 lA, 360° rotation, and a pixel size of 13.73 mm. Three-dimensional reconstruction and analysis were performed using Mimics Research software (v19.0, Materialise, Leuven, Belgium), generating 1000–1200 axial slices per specimen. Micro-CT grayscale values were analyzed within a standardized 4-mm segment of the medium-sized fiber posts, excluding the apical 1-mm portion. Material segmentation was achieved through calibrated thresholding of: (1) dentin, (2) resin cement, and (3) voids, with fiber posts identified by uniform density.

**Fig 1:**
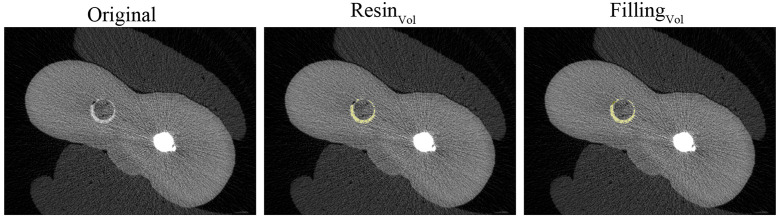
Representative and consecutive micro-CT cross sections from the medium 4-mm post. The original grayscale images revealed the presence of voids (depicted as black spots within the resin) and resin (highlighted in white). The Resin_Vol_ (coloured in yellow) was calculated, and the Filling_Vol_ encompassed both the Voids_Vol_ and the Resin_Vol_.

The established resin cement grayscale thresholds enabled both qualitative and quantitative volumetric assessments. For each axial slice (Fig 1), the region of interest was defined as the total filling volume, comprising both resin cement and void spaces. Two calibrated evaluators, blinded to experimental groups, independently analyzed all datasets. Void volume (Voids_Vol_) was mathematically derived by subtracting the resin cement volume (Resin_Vol_) from the total filling volume (Filling_Vol_) according to the equation: Voids_Vol_ = Filling_Vol_ – Resin_Vol_.

The percentage volume of the voids (%Voids_Vol_) was calculated via the following formula: %Voids_Vol_ = Voids_Vol_ *100/Filling_Vol_ [15].

### Push-out test

**Fig 2:**
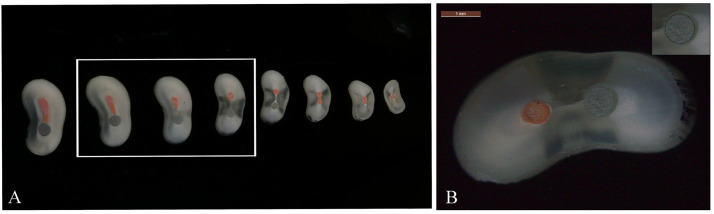
(A. B) Images of the slices and the medium portion marked for calculation of the radius of the fiber posts.

The specimens were transversely sectioned perpendicular to the root’s longitudinal axis using a precision low-speed saw (Accutom-50, Struers Ltd., Ballerup, Denmark) under continuous water cooling to minimize thermal damage. Slices containing the fiber posts were collected, discarding the first and last slices, resulting in approximately 3 slices per sample (Fig 2A). The depths of the slices within the roots were categorized as follows: I, apical; II, medium; and III, cervical.

Both surfaces (cervical and apical aspects) of each section were imaged using a calibrated optical microscope (DM750, Leica Microsystems, Wetzlar, Germany). Post diameters (ImageJ, National Institutes of Health, Bethesda, MD; http://imagej.nih.gov/ij/) and slice thicknesses (digital micrometer, Vernier Caliper; AIRAJ, China) were measured from 20 × microscope images (Fig 2B).

Bond strength evaluation was performed using a universal testing machine (Instron Co. Ltd., USA). A constant crosshead speed of 0.5 mm/min was applied in the apical-coronal direction until failure occurred (post dislodgement). The maximum load at failure was recorded in Newtons (N). The bond strength values (MPa) were calculated as the load (N) divided by the bonded surface area (S). The bonded surface area (S) of each tapered post segment was calculated as: S = π(R + r) [h^2^+ (R − r)^2^]^0.5^, where R and r are coronal and apical post respectively, and h is section thickness.

### Failure mode analysis

**Fig 3:**
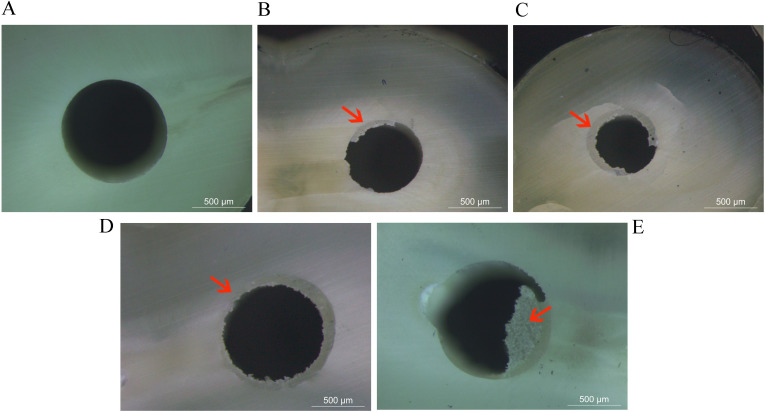
Failure modes characterized by fracture location (arrows), as evaluated via optical microscopy. (A) Adhesive failure between dentin and resin cement, (B) mixed failure with resin cement covering 0-50% of the dentin, (C) mixed failure with resin cement covering 50-100% of the dentin, (D) adhesive failure between the post and resin cement, and (E) cohesive failure within the post/dentin.

Failure modes were characterized using a high-resolution optical microscope (DM750, Leica Microsystems, Wetzlar, Germany) at 60 × magnification. The mode of failure was categorized into five types: (I) adhesive failure between dentin and resin cement, (II) mixed failure with resin cement covering 0–50% of the dentin, (III) mixed failure with resin cement covering 50–100% of the dentin, (IV) adhesive failure between the post and resin cement, and (V) cohesive failure within the post/dentin (Fig 3) [16].

### Statistical analysis

The data were analysed via SPSS software (Version 20, Chicago, IL, USA). The length, %Voids_Vol_ of the fiber posts and bond strength, as confirmed by the Shapiro‒Wilk test, exhibited a normal distribution. One-way analysis of variance (ANOVA) and the Bonferroni correction tests were employed for statistical analysis. A *P* value less than 0.05 was considered statistically significant.

## Results

Table 1 shows the mean percentages and standard deviations of the length values (mm) of the 4 groups in all directions. No significant differences were observed (P > 0.05), indicating that the shape of the samples in each group was balanced and comparable.

**Table 1:** Mean percentages and standard deviations of all direction length values (mm) of the 4 groups.

Group	Root length	Bucco-lingual diameter	Proximal‒distal diameter
SC-I	15.03 ± 1.01	4.86 ± 0.32	8.42 ± 0.33
CWC- I SC-D	15.27 ± 0.82 15.77 ± 0.47	4.91 ± 0.28 4.97 ± 0.37	8.63 ± 0.55 8.75 ± 0.40
CWC-D	15.10 ± 0.97	5.01 ± 0.24	8.78 ± 0.51

**Fig 4:**
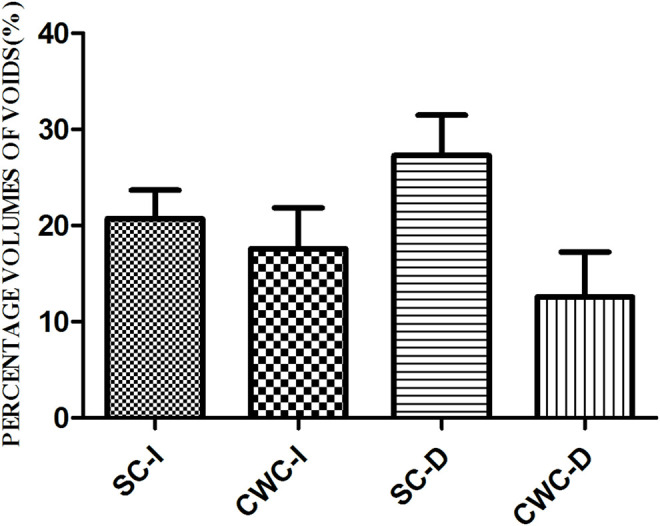
%Voids_Vol_ (%, mean±standard deviation) for each cross-sectional root canal of the middle portion of the post in each group.

Fig 4 shows the mean (mean±standard deviation) %Voids_Vol_ of the medium 4-mm fiber posts among the groups. No significant differences were observed (P > 0.05), indicating that the post cementation of the fiber in each group was consistent and achieved the standardization of the samples for the PBS experiments. The %Voids_Vol_ of the fiber posts was similar and was not significantly influenced by the timing of post-space preparation or the obturation technique.

**Fig 5:**
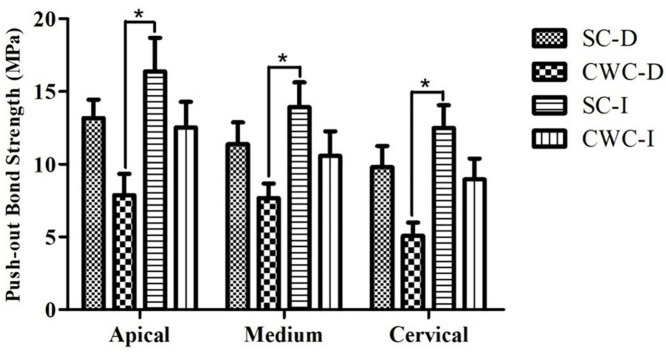
Bond strength (MPa, mean±standard deviation) for each cross-sectional root canal of the post in each group. From the apical to the cervical depths, the bond strength was greater when the single-cone technique followed by immediate post-space preparation was used than when the continuous wave of condensation followed by delayed post-space preparation was used (*P < 0.05).

In the PBS experiment, the bond strength values of the immediate fiber posts cemented with SC were greater than those of the delayed fiber posts cemented with CWC, with no significant differences observed among the different post depths (Fig 5). Moreover, adhesive failure between the dentin and the resin cement was the most prevalent fracture mode observed (Table 2).

**Table 2:** Failure modes (%) observed for the post in each group.

Group	1	2	3	4	5
Apical					
SC-D	56.56	33.33	–	11.11	–
CWC-D	66.64	9.09	18.18	9.09	–
SC-I	70.00	20.00	10.00	–	–
CWC-I	60.00	40.00	–	–	–
Medium					
SC-D	40.00	20.00	10.00	10.00	20.00
CWC-D	60.00	40.00	–	–	–
SC-I	36.36	45.45	18.18	–	–
CWC-I	70.00	30.00	–	–	–
Cervical					
SC-D	30.00	40.00	10.00	20.00	–
CWC-D	70.00	–	20.00	10.00	–
SC-I	54.55	36.36	–	–	9.09
CWC-I	60.00	40.00	–	–	–

1: Adhesive failure between dentin and resin cement

2: Mixed failure with resin cement covering 0–50% of the dentin

3: Mixed failure with resin cement covering 50–100% of the dentin

4: Adhesive failure between the post and resin cement

5: Cohesive failure within the post/dentin

**Fig 6:**
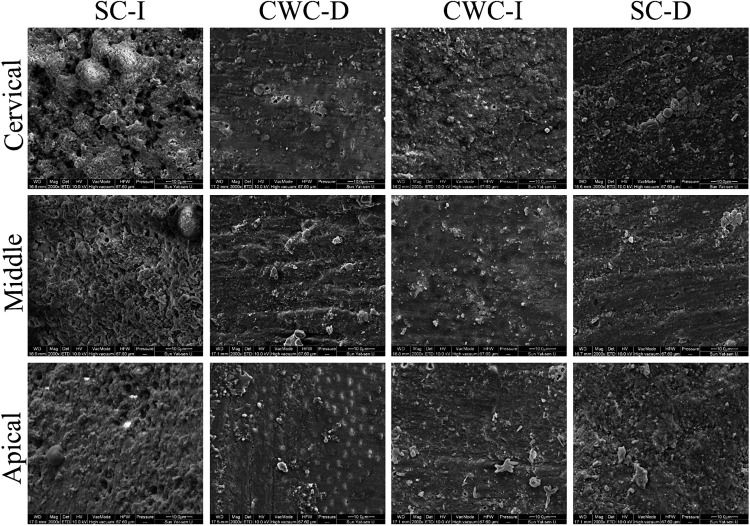
Photomicrographs of the cervical, middle and apical regions after post-space preparation. The smear layer was fully visible, and the dentin tubule mouths were nearly closed in the apical, middle, and cervical regions of the samples. SC-I significantly reduced the smear layer on the root dentin wall.

SEM examination of the root dentin wall revealed a visible smear layer and nearly closed dentine tubule mouths in the apical, middle, and coronal regions of the samples. Partial removal of the smear layer was observed in the SC-I group, with visible outlines of dentine tubules in the apical, middle, and cervical regions of the samples (Fig 6).

## Discussion

Endodontically treated teeth have undergone extensive loss of coronal structure and lack adequate support for permanent restoration. In these cases, additional retention from the root canal may be needed [17]. Thus, these teeth may need core retention through intracanal post placement [18,19]. Modern endodontic practice advocates conservative canal preparation and prompt adhesive coronal restoration to minimize risks of vertical root fracture and bacterial microleakage [20].

Fiber posts have gained widespread acceptance in contemporary endodontics as a superior alternative to conventional cast posts, owing to their favorable biomechanical properties and clinical performance [2,21]. Fiber posts demonstrate three principal advantages in endodontic restoration: (1) an elastic modulus closely approximating that of dentin, (2) superior optical characteristics that mimic natural tooth structure, and (3) the capacity for adhesive cementation that creates a unified monobloc structure through molecular bonding at the dentin-resin-post interfaces [3,4,22]. This adhesive restorative protocol is critical for achieving long-term clinical success, as it establishes a durable interfacial bond between the fiber post, resin cement, and dentin – a fundamental requirement for the functional rehabilitation of endodontically treated teeth.

Post-space preparation is commonly classified as immediate or delayed; however, a consensus on the optimal timing remains elusive in clinical practice. The traditional approach favours delayed post-space preparation, which is usually performed 1–2 weeks after root canal filling, allowing time for treatment observation and material solidification [23]. However, some argue that issues such as recurrent caries, open crown margins, leakage through temporary restorations, or the absence of timely provisional restorations can significantly contribute to coronal leakage, a major factor in root canal failure. Thus, immediate post-crown repair becomes essential when there is a clear need for core retention [24].

Our experimental results indicate that the timing of post space preparation (whether immediate or delayed) did not significantly affect the bond strength when either the single-cone technique or continuous wave condensation technique was employed. Nevertheless, the clinical advantages of immediate post space preparation remain evident. First, immediate post preparation can be carried out under a rubber dam, maintaining consistent aseptic conditions without the need to reopen the root canal, as seen in delayed post preparation. This significantly reduces the risk of microbial infection. Concurrently, the operator’s familiarity with the root canal system allows for precise assessment and potential improvement of the remaining apical gutta-percha. Current research suggests that the post length should extend to approximately two-thirds of the total root length [25]. However, achieving this ideal length can be challenging when balancing the need for an adequate ferrule effect and maintaining apical sealing integrity. To ensure a reliable apical seal, a minimum of 3−5 mm of gutta-percha should be preserved at the apical region. Additionally, the post diameter must not exceed one-third of the root diameter at any point to avoid structural compromise [26,27]. Careful consideration of root dimensions is essential, as improper post space preparation may lead to apical or lateral root perforation. By utilizing the obturation unit, the post space can be mechanically prepared with a post drill after thermally removing the coronal root filling material, minimizing the impact of P or G drills on the sealing of the apical root canal filling material. Heat-assisted removal of filling material offers greater efficiency and safety compared to mechanical methods. Since drills act as the final cutting instrument, excessive force or improper angulation can lead to dentinal gauging, eccentric preparation, or even root perforation. The hot plugger method helps avoid deviations from canal anatomy, minimizing the risk of perforation or stripping, especially during immediate post-space preparation. Finally, immediate post-space preparation streamlines the operation and shortens treatment duration [28,29].

Aligning with our results [30], Ozkurt-Kayahan et al. also found no significant difference in apical microleakage between the SC and calamus techniques following immediate post-space preparation [31]. However, there is disagreement regarding the time interval between root canal filling and post-space preparation. A study revealed that the shortest initial setting of a bioceramic root canal sealer and an epoxy resin-based sealer (AH Plus) varies from 1–8 hours, and the final setting time varies from 11–24 hours, which is longer than that reported by manufacturers [32]. Therefore, the time of post-space preparation may influence some outcomes in endodontically treated teeth, such as void formation [33], displacement of the filling material [34], and postoperative pain [35].Contrary to our findings, Schäfer et al. found that delayed post-space preparation compromised obturation quality with the SC technique but had no significant effect with the CWC technique [36]. Regarding the effects of immediate versus delayed post-space preparation on the apical sealing ability of the SC and CWC techniques, Dalat and Spångberg proposed that root filling material could be dislodged during mechanical post-space preparation if the sealer has not fully set [21]. In contrast, there was no gutta-percha to dislodge, and the %Voids_Vol_ of the apical filling material showed no significant differences [30]. However, a clinical study revealed that the pain intensity score was significantly greater in the immediate post-space preparation group than delayed post-space preparation group. The vibrations produced during simultaneous post placement and root canal obturation may increase postoperative pain. Current evidence shows no consistent association between treatment visit frequency (single- versus two-visit) and postoperative pain following endodontic restoration [35].

An ideal endodontic sealer should fulfil all ideal requirements, and an epoxy resin-based sealer (AH Plus) is used as the gold standard in research because of its excellent properties related to adhesion and sealing ability [37,38]. However, Kaul et al. [39] observed that the AH Plus group presented greater leakage values between the gutta‑percha and the dentin wall than did the two groups of bioceramic sealers. Bioceramic sealers are relatively new materials that are available. Iroot SP, a bioceramic root canal sealer, primarily comprises calcium silicate and calcium phosphate, facilitates the formation of hydroxyapatite during its setting process [23]. While meeting all required chemical and physical standards, the premixed bioceramic sealers demonstrated greater open pore volume, water absorption, and solubility than traditional epoxy resin-based sealers [40]. In addition, the bioceramic sealer can generate tag-like structures composed of crystals, which precipitate within the intratubular space and remain within the dentinal tubules [41]. Consequently, it becomes challenging to clean the intratubular space after complete solidification, hindering resin cement penetration inside the tubules and impeding the formation of an effective hybrid layer with resinous tags [41]. For instance, Vilas-Boas et al. [42] evaluated the push-out bond strength of fiber posts and found that the EndoSequence BC sealer exhibited significantly lower bond strength than AH Plus, irrespective of the cementation time. This difference was attributed to the substantial amount of residual BC sealer remaining within the dentinal tubules following post-space preparation. However, in the present experiment, the timing of post space preparation showed no significant effect on push-out bond strength. Theoretically, early removal of unset sealer should have resulted in higher bond strength in the immediate preparation groups. Yet, no significant differences were observed between immediate and delayed post space preparation groups, regardless of whether the single-cone technique or continuous wave compaction technique was used. This phenomenon may be attributed to the following reasons: First, complete removal of unset sealer cannot be achieved [42,43]; Second, the alkaline by-products generated during the setting reaction may interfere with the acidic bonding agents of resin cements [44]; Third, the final products of calcium silicate-based sealer hydration and precipitation-hydroxyapatite and water [45]-may complexly affect the interfacial bonding. Specifically, water molecules produced by this reaction could disrupt the polymerization of hydrophobic resin cements [46]. Currently, there is limited evidence regarding the potential interference of bioceramic sealers with the bond strength of fiber posts cemented using resin-based cements. Further experimental studies are required to elucidate this issue.

In our PBS experiment, the SC with immediately cemented fiber posts presented higher bond strength values than did the CWC with delayed cemented fiber posts. This difference may be attributed to the relatively effective removal of residues and the smear layer within the root canal dentin tubules, as SEM analysis revealed visible outlines of dentine tubules with partial smear layer removal in the apical, middle, and cervical regions of samples from the SC-I group. The observed effect may be partly due to the higher percentage of voids in the cervical third region reported in SC techniques compared to CWC [2]. Furthermore, relative to warm vertical compaction techniques, the SC technique tends to result in less favorable root canal filling quality, particularly in curved canals where greater sealer accumulation has been observed [3]. The increased volume of sealer in these areas renders the polar regions of the root filling more vulnerable to disruption under mechanical stress, consequently reducing bond strength to dentin [4]. As a result, filling materials can be more readily removed from the post space when using the SC technique compared to CWC, especially when sealer has not fully set during immediate post-space preparation.

Micro-CT has been utilized for three-dimensional analysis to measure voids and assess the quality of fiber post cementation because of its effectiveness, noninvasiveness, and nondestructive nature [47]. In this study, there were no significant differences observed in the %Voids_Vol_ of the fiber post results. The absence of statistically significant differences in porosity among groups demonstrated that the post space preparation and cementation procedures achieved standardized requirements across different preparation timings, thereby establishing a reliable experimental foundation for subsequent push-out bond strength testing. During post-space preparation, the drills generated a smear layer rich in gutta-percha, root canal sealant, and inorganic components. The procedure for post-space preparation was meticulously conducted and standardized across all groups to eliminate excess endodontic filling material from the prepared root canal space. However, remnants of the smear layer and debris along the post space canal walls were located at the entrance of the dentinal tubules even after post-space preparation and cleaning. Our SEM examination of the root dentin wall revealed a visible smear layer and nearly closed dentine tubule mouths in the apical, middle, and coronal regions of the samples. This could alter the wettability, permeability, and reactivity of the dentin, impede adhesive resin penetration, and compromise the bond strength of fiber posts to dentin [42,48,49]. Consistent with these observations, the predominant failure mode in this study was adhesive failure at the dentin-resin cement interface, with minimal failure observed at the post-resin cement interface. These findings underscore that thorough removal of the smear layer is critical for achieving reliable bonding between fiber posts and intraradicular dentin [50,51].

However, it is challenging to directly extrapolate in vitro conditions to actual clinical situations, where teeth experience compressive occlusal loads along with shear and bending forces. Studies utilizing pull-out tests are considered a more substantial methodology for analysing the bond strength between fiber posts and root canal dentin [52,53]. Nonetheless, this study has several limitations, including the inability to standardize the extracted teeth during sample preparation, the exclusive use of the PBS test for bond strength evaluation, variations in post space preparation, fiber post cementation, and surface treatment applications, as well as inconsistencies in operator performance during these procedures. Therefore, further clinical research is essential to validate the current findings.

## Conclusion

The present study demonstrated that SC followed by immediate post-space preparation provided better bond strength of fibre posts to intraradicular dentine than did CWC followed by delayed post-space preparation. These results revealed that the choice of the obturation technique and the time of post-space preparation may influence the bond strength of the fiber posts to the intraradicular dentin
